# Remarkable response to fluorouracil, leucovorin, oxaliplatin, and irinotecan therapy in urothelial cancer of the renal pelvis: a case report

**DOI:** 10.1186/s13256-017-1263-x

**Published:** 2017-04-10

**Authors:** Takuya Tsujino, Kiyoshi Takahara, Tomohisa Matsunaga, Yuki Yoshikawa, Tomoaki Takai, Taizo Uchimoto, Kenkichi Saito, Naoki Tanda, Hajime Hirano, Hayahito Nomi, Naokazu Ibuki, Teruo Inamoto, Haruhito Azuma

**Affiliations:** Department of Urology, Osaka Medical College, 2-7 Daigaku-cho, Takatsuki, Osaka 569-8686 Japan

**Keywords:** Urothelial carcinoma, Upper tract urothelial carcinoma, FOLFOXIRI, Oxaliplatin, Fluorouracil, Irinotecan, Case report

## Abstract

**Background:**

No standard chemotherapy regimen for advanced urothelial cancer has been established, except for cisplatin-based regimens. We report the case of a patient with double primary cancer, urothelial carcinoma of the upper urinary tract and colorectal cancer, who underwent oxaliplatin-based chemotherapies.

**Case presentation:**

A 56-year-old Japanese man presented to our hospital with the diagnosis of a left renal pelvic tumor and rectal cancer. Several examinations including ureteroscopic biopsy and computed tomography-guided biopsy were performed; however, the diagnosis of renal pelvic cancer could not be made. Because the rectal cancer had been growing during the course of examination, he underwent five cycles of neoadjuvant chemotherapy with fluorouracil, leucovorin, oxaliplatin, and irinotecan. The volumes of both the rectal cancer and renal pelvic tumor drastically decreased. He then underwent pelvic evisceration with colostomy and ureterocutaneostomy. The histological diagnosis of the renal pelvic tumor was urothelial carcinoma. He is free of disease at 12 months after the treatment.

**Conclusions:**

To the best of our knowledge, this is the first report describing a remarkable response to fluorouracil, leucovorin, oxaliplatin, and irinotecan therapy for renal pelvic cancer. We suggest fluorouracil, leucovorin, oxaliplatin, and irinotecan is an effective therapy for patients with advanced urothelial cancer.

## Background

Most cases of urothelial carcinoma (UC) develop in the urinary bladder, while UC of the upper urinary tract (UTUC) is uncommon, accounting for only 5 to 10% of all renal tumors. Radical nephroureterectomy with bladder cuff excision remains the standard treatment for patients with UTUC with large, multifocal or high-grade tumors. The effect of perioperative chemotherapy for UTUC remains unclear. We report the remarkable anti-tumor effect of oxaliplatin-based chemotherapies for UTUC.

## Case presentation

**Fig. 1:**
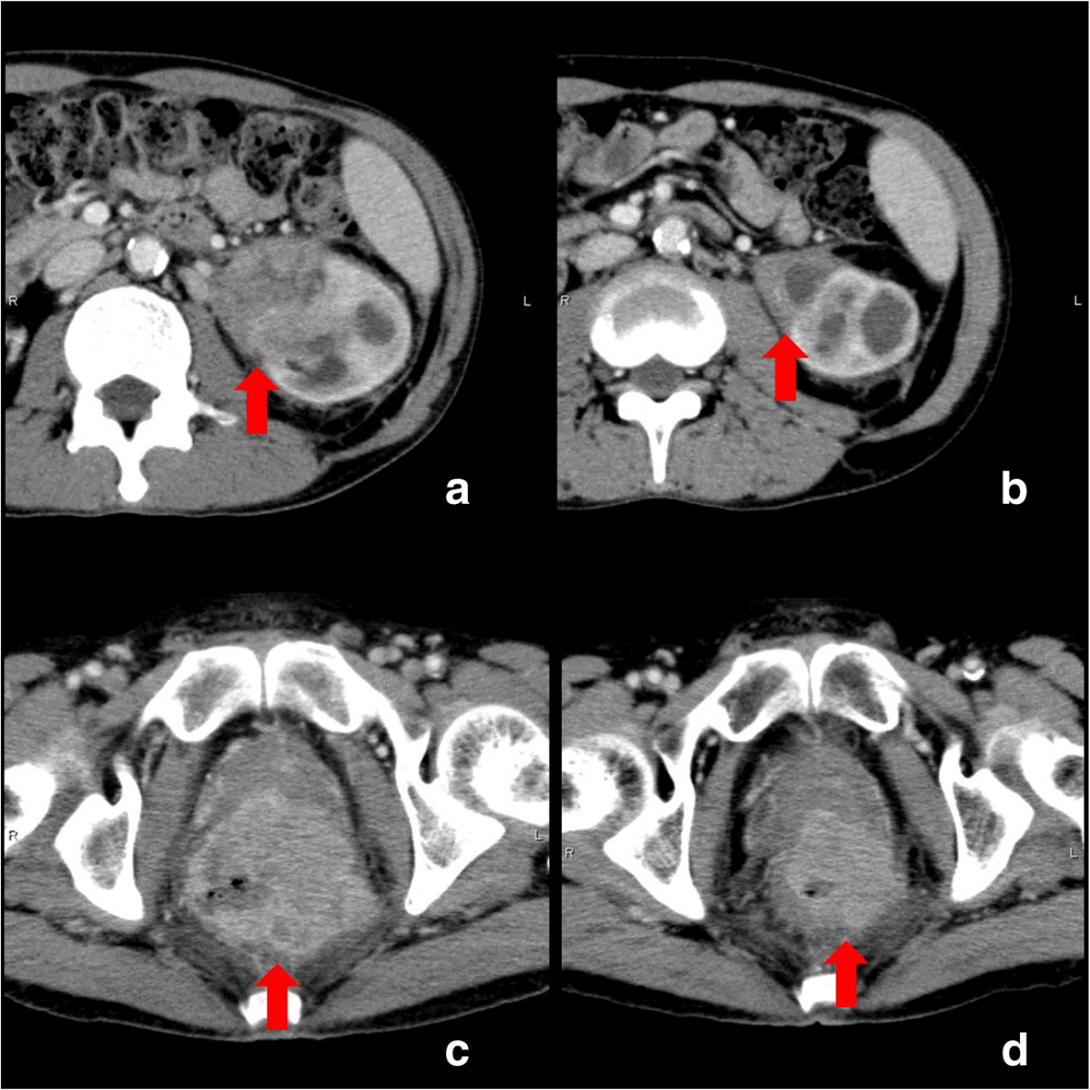
**a**, **b** Abdominal computed tomography scan demonstrating the volume of renal pelvic tumor before treatment (**a**), and the decrease in volume after two cycles of fluorouracil, leucovorin, oxaliplatin, and irinotecan (**b**). **c**, **d** The rectal cancer before treatment (**c**), and decrease in tumor volume after two rounds of fluorouracil, leucovorin, oxaliplatin, and irinotecan (**d**). *Red arrows* indicate the sites of the tumor

**Fig. 2:**
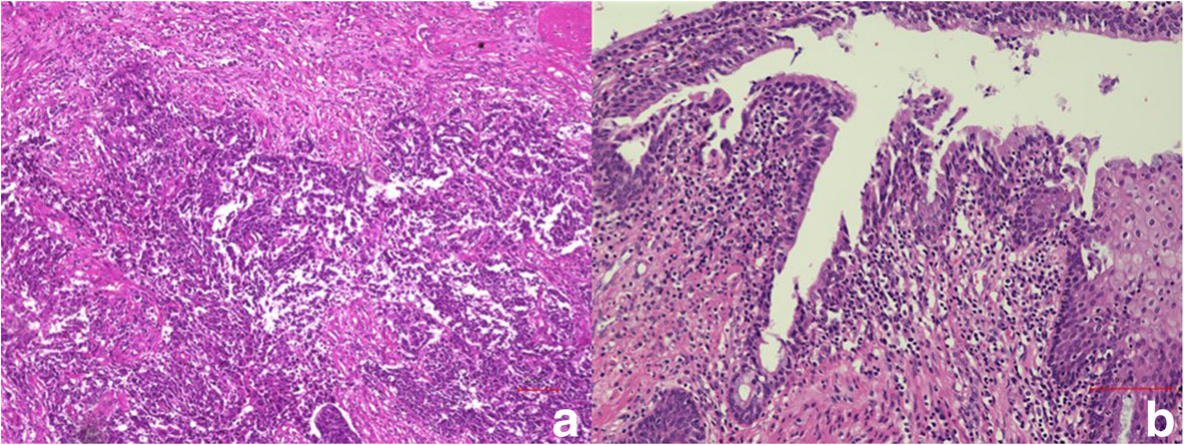
**a** The renal pelvis tumor was histologically diagnosed as high-grade urothelial carcinoma (hematoxylin and eosin, ×100). **b** The rectal cancer revealed adenocarcinoma (hematoxylin and eosin, ×200)

A 56-year-old Japanese man with a diagnosis of a left renal pelvic tumor and rectal cancer was referred to our hospital. He had been smoking cigarettes for 36 years. He had been healthy all his life and his family history was unremarkable. His chief complaints were bloody stools and proctodynia for several months. On admission, his vital signs were normal. An abdominal examination revealed distension; a rectal examination showed bloody stool and narrowing of his anus. The findings on other examinations were completely unremarkable. Laboratory examination revealed anemia and an inflammatory response (hemoglobin level of 100 g/L, white blood cell count of 12.5×10^9^/L, neutrophil count of 10.8×10^9^/L, and lymphocyte count of 1.3×10^9^/L); the other findings were normal. His level of carcinoembryonic antigen (CEA) was normal. Contrast-enhanced abdominal computed tomography (CT) showed a huge mass in his rectum and a left renal pelvic tumor. Histological examination of a sample obtained during colonoscopy revealed adenocarcinoma of the rectum. Several examinations including ureteroscopic and CT-guided biopsies were performed; however, the diagnosis of renal pelvic cancer could not be made. Because the rectal cancer was growing during our evaluations of the patient, we started to administer two cycles of neoadjuvant chemotherapy with fluorouracil, leucovorin, oxaliplatin, and irinotecan (FOLFOXIRI). The volumes of the rectal cancer and renal pelvic tumor decreased drastically at the end of the two cycles of chemotherapy (Fig. 1). He received a total of five cycles of FOLFOXIRI. The renal pelvic tumor reduced by 50% as measured across the maximum diameter (from 64 mm to 32 mm). During the administration of chemotherapy, laboratory examination revealed neutropenia (white blood cell count of 1.7×10^9^/L with 0.46×10^9^/L neutrophils). Febrile neutropenia was observed at the end of the first round of chemotherapy; however, no other severe adverse events were observed after that. Subsequently, laparoscopic total nephroureterectomy on the left side was performed following pelvic evisceration with colostomy and ureterocutaneostomy on the right side. On histological examination, the rectal cancer and renal pelvic tumor were diagnosed as adenocarcinoma (pT2N0M0) and UC (pT3N0M0) with high grade, respectively, as presented in Fig. 2. There has been no disease progression at 12 months after treatment.

## Discussion

UC responds very well to cisplatin-based chemotherapy, represented by gemcitabine-cisplatin (GC) therapy; therefore, this treatment is widely administered to patients with UC. However, effective chemotherapy regimens, other than cisplatin-based chemotherapies, for patients with UC have not been established, although many clinical trials are ongoing. Oxaliplatin is more effective than cisplatin *in vitro*, and has shown efficacy in preclinical studies using many tumor cell lines [1]. In addition, Siew *et al*. demonstrated the efficacy of oxaliplatin, an immunogenic cell death inducer, on the induction of stress ligands and promotion of natural killer cell-mediated cytotoxicity in human ovarian cancer [2]. The efficacy of an oxaliplatin alone chemotherapy for advanced or metastatic UC was minimal in phase II studies. Those studies showed response rates of not more than several percent [3, 4]. The efficacy of 5-fluorouracil (5-FU) for advanced UC remains unclear, but a review of published studies in 1987 described response rates of approximately 15% using the unmodulated single agent 5-FU [5]. In addition, a phase II trial of continuous 5-FU infusion in 2009 showed a median progression-free survival of 1.9 months, a median overall survival of 6.5 months, and a response rate of 20% [6].

In 2008, a phase II study was performed to determine the efficacy of irinotecan monotherapy for advanced transitional cell carcinoma of the urothelium; the median progression-free survival was 2.1 months and the median overall survival was 5.4 months [7]. However, these monotherapy regimens do not usually give good responses for cancers, including colorectal cancer. The response rates of irinotecan, oxaliplatin, and 5-FU were only approximately 18%, 20%, and 21%, respectively. On the other hand, combination therapies using a combination of fluorouracil, leucovorin, and irinotecan (FOLFIRI), a combination of fluorouracil, leucovorin, and oxaliplatin (FOLFOX), and FOLFOXIRI had stronger activities in colorectal carcinoma [8–11]. There are some reports on the use of combination therapies for patients with UC. The case report about the use of FOLFIRI for UC by Lu *et al*. showed that UTUC was well controlled by the chemotherapy regimen, and, that it was effective for metastatic colorectal cancer [12]. There are few reports of FOLFOX therapy for UC, with only a phase II trial by Lorenzo *et al*. [13]. They used FOLFOX in 18 patients who had previously been treated for UC, and reported only low-grade toxicity and a 19% overall response rate [13]. The case report of FOLFOX for UC by Seo *et al*. showed a complete response in a patient who developed lung metastasis and an additional primary colon cancer after radical nephrectomy for the UC [14]. In our patient with UC, chemotherapy with FOLFOXIRI resulted in an extremely good response.

## Conclusions

To the best of our knowledge, this is the first report describing a remarkable response to FOLFOXIRI therapy in a renal pelvic cancer and UC. Therefore, we suggest FOLFOXIRI therapy is a novel and effective therapy for advanced UC patients.
